# Deep learning-based detection of dental prostheses and restorations

**DOI:** 10.1038/s41598-021-81202-x

**Published:** 2021-01-21

**Authors:** Toshihito Takahashi, Kazunori Nozaki, Tomoya Gonda, Tomoaki Mameno, Kazunori Ikebe

**Affiliations:** 1 Department of Prosthodontics, Gerodontology and Oral Rehabilitation, Osaka University Graduate School of Dentistry, 1-8 Yamadaoka, Suita, Osaka 565-0871 Japan; 2 Division of Medical Information, Osaka University Dental Hospital, 1-8 Yamadaoka, Suita, Osaka 565-0871 Japan

**Keywords:** Dentistry, Diagnosis

## Abstract

The purpose of this study is to develop a method for recognizing dental prostheses and restorations of teeth using a deep learning. A dataset of 1904 oral photographic images of dental arches (maxilla: 1084 images; mandible: 820 images) was used in the study. A deep-learning method to recognize the 11 types of dental prostheses and restorations was developed using TensorFlow and Keras deep learning libraries. After completion of the learning procedure, the average precision of each prosthesis, mean average precision, and mean intersection over union were used to evaluate learning performance. The average precision of each prosthesis varies from 0.59 to 0.93. The mean average precision and mean intersection over union of this system were 0.80 and 0.76, respectively. More than 80% of metallic dental prostheses were detected correctly, but only 60% of tooth-colored prostheses were detected. The results of this study suggest that dental prostheses and restorations that are metallic in color can be recognized and predicted with high accuracy using deep learning; however, those with tooth color are recognized with moderate accuracy.

## Introduction

When planning the dental treatment plan for each patient, to grasp the intraoral information is necessary whatever the treatment is. In clinical practices, this procedure is conducted by dentist and it often takes time. Furthermore, the result of this procedure depends on a dentist’s knowledge and experience. Therefore, there is a need for an automated system for grasping the intra-oral situation in the short time.

In recent years, artificial intelligence (AI) technologies have been applied in dental medicine, obviating the need for human input in some cases. For instance, AI-based methods and clinical applications have been developed for the diagnosis of dental caries^1,2^ and oral cancer^3^ with an accuracy that is comparable to that of human beings. These applications have involved the use of deep learning, which is an object detection method that makes predictions given various images of objects. The intraoral information consists of the missing area and occlusion, the shape and location of the residual teeth, restorative or prosthetic situation, periodontal or endodontic situation, risk of caries or periodontal disease, and so on. In addition to above applications, evaluation of periodontal^4,5^ or endodontic situation^6^ using deep learning were already reported. In our previous report, classification of the missing area was accomplished with a high prediction rate^7^. However, evaluation of prosthodontic situation was not conducted yet. In this study, the recognition of prosthodontic situation, that is dental prostheses and the restoration of residual teeth, both types and materials, was performed using deep learning.

The aim of this study was to recognize of dental prostheses and restorations using a deep-learning object detection method.

## Results

**Figure 1:**
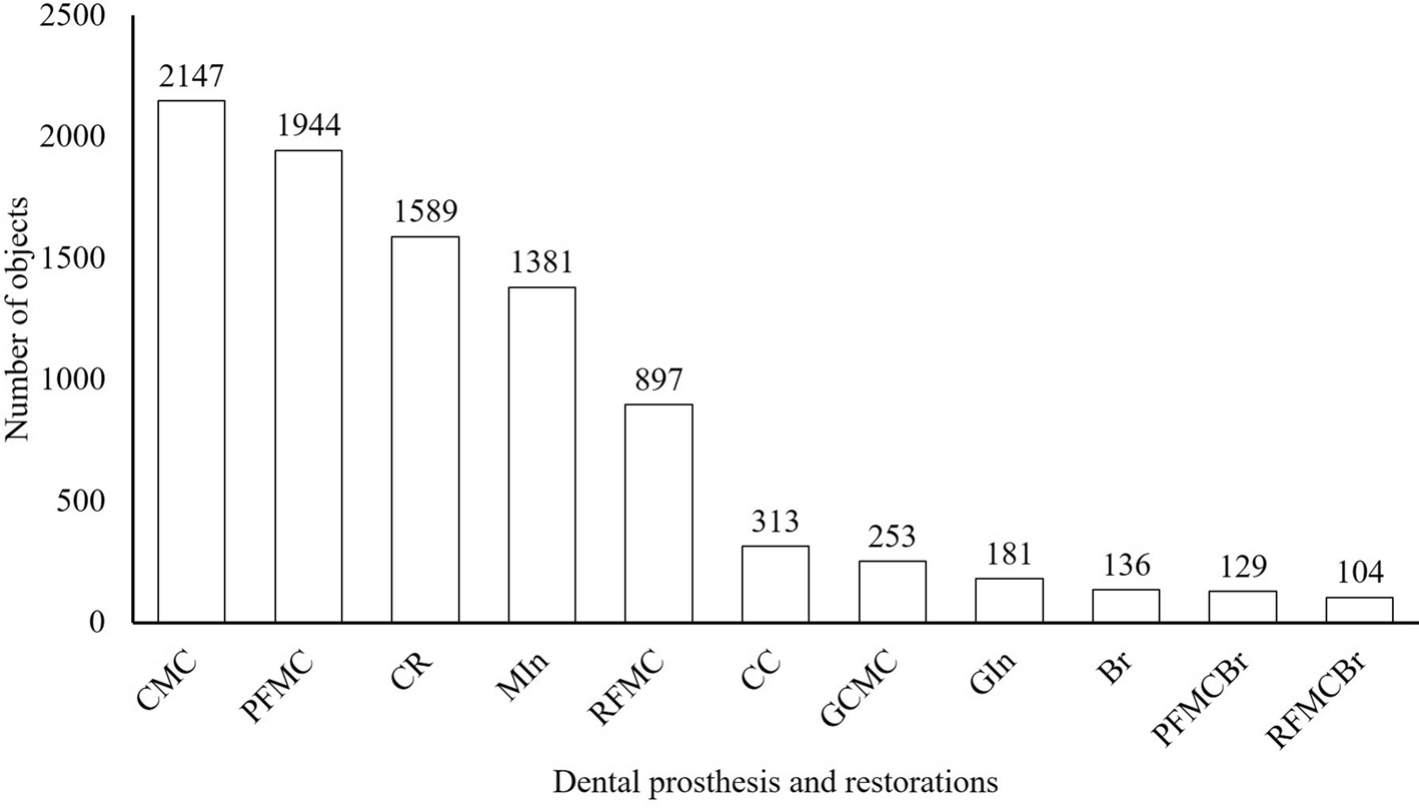
Total number of objects of dental prostheses and restorations in all images. CMC: silver-colored complete metal crown, PFMC: porcelain fused to metal crown, CR: composite resin filling, MIn: silver-colored metal inlay restoration, RFMC: resin facing metal crown, CC: ceramic crown, GCMC: gold-colored complete metal crown, GIn: gold-colored metal inlay restoration, Br: fixed partial denture of CMCs, PFMCBr: fixed partial denture of PFMCs, and RFMCBr: fixed partial denture of RFMCs.

**Figure 2:**
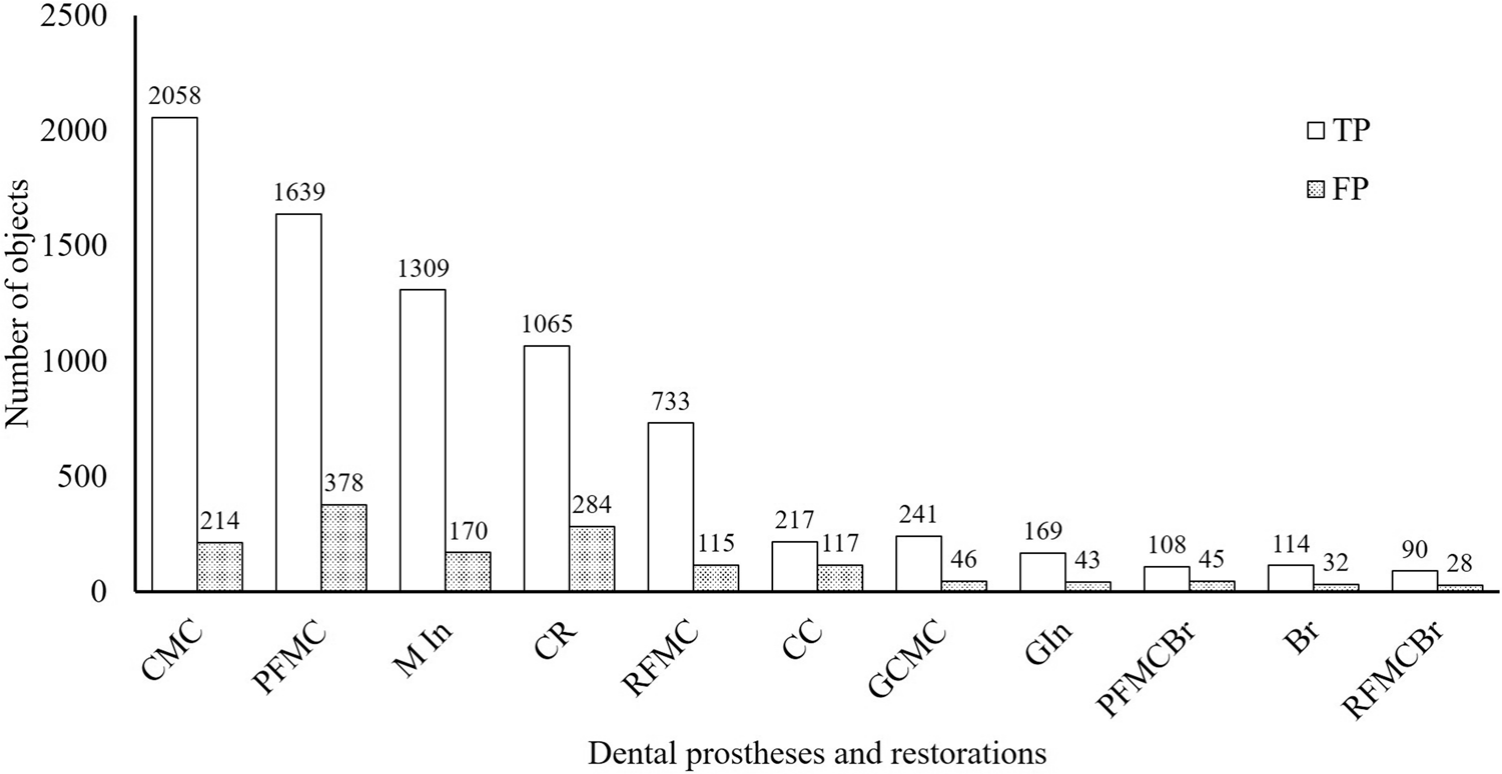
Total number of dental prostheses and restorations detected correctly (TP) and those detected as other prostheses (FP). CMC: silver-colored complete metal crown, PFMC: porcelain fused to metal crown, MIn: silver-colored metal inlay restoration, CR: composite resin filling, RFMC: resin facing metal crown, CC: ceramic crown, GCMC: gold-colored complete metal crown, GIn: gold-colored metal inlay restoration, RFMCBr: fixed partial denture of RFMCs, Br: fixed partial denture of CMCs, and PFMCBr: fixed partial denture of PFMCs.

**Figure 3:**
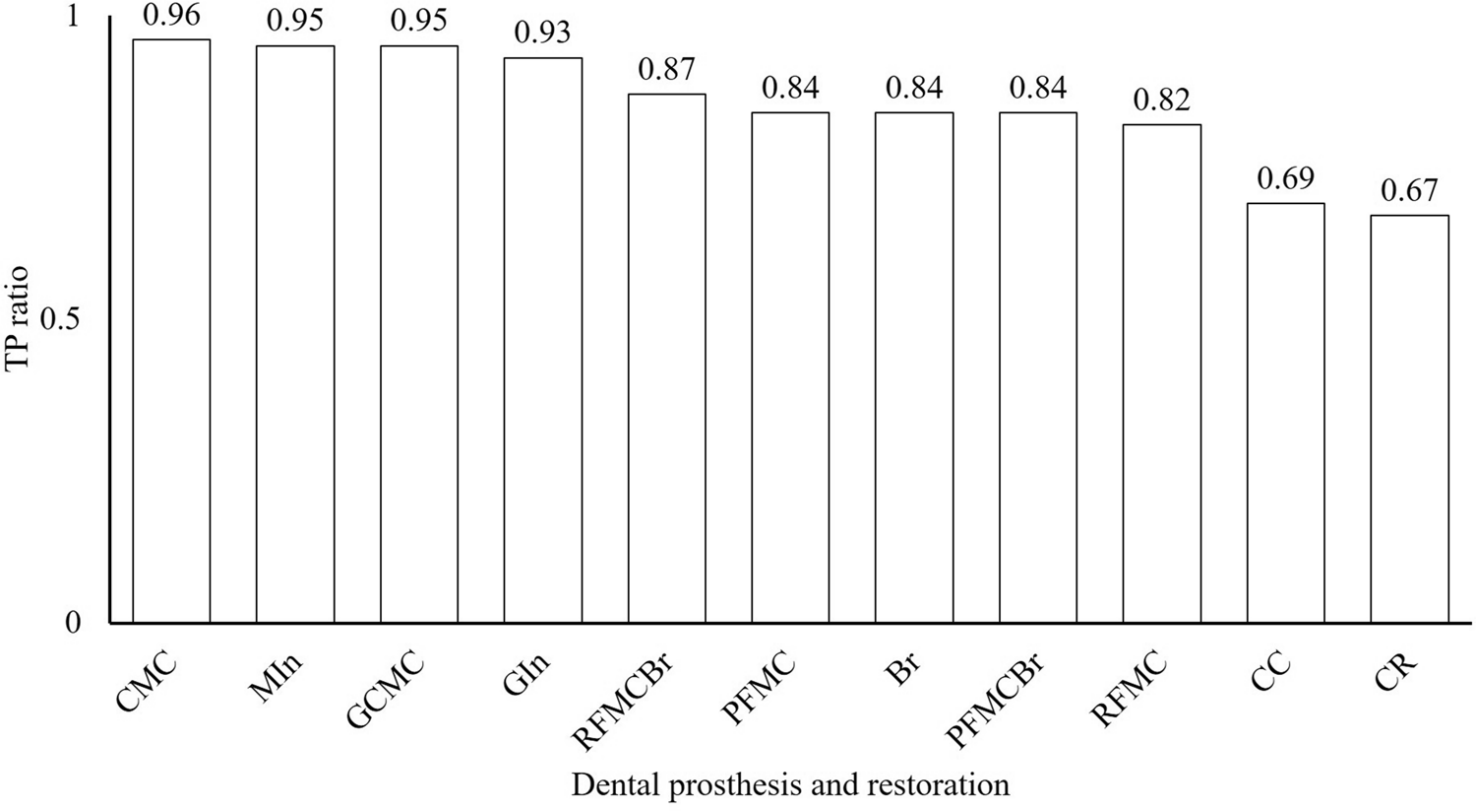
Ratio of dental prostheses detected correctly (TP) to all detected prostheses. CMC: silver-colored complete metal crown, MIn: silver-colored metal inlay restoration, GCMC: gold-colored complete metal crown, GIn: gold-colored metal inlay restoration, RFMCBr: fixed partial dentures of RFMCs, PFMC: porcelain fused to metal crown, Br: fixed partial denture of CMCs, PFMCBr: fixed partial denture of PFMCs, RFMC: resin facing metal crown, CC: ceramic crown, and CR: composite resin filling.

**Figure 4:**
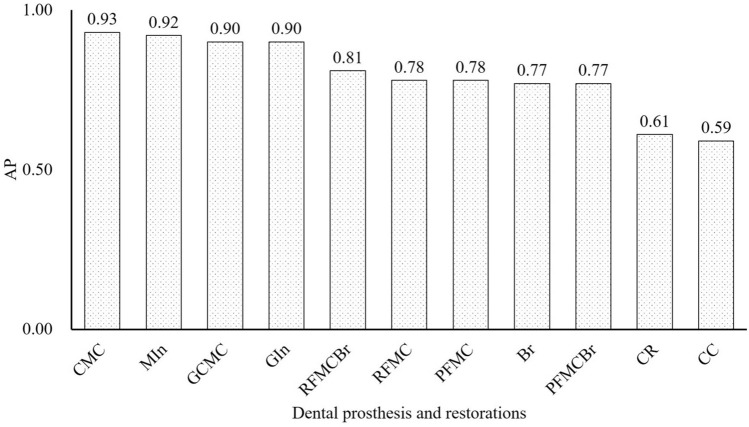
Average precision (AP) of each dental prosthesis and restoration in all images. CMC: silver-colored complete metal crown, MIn: silver-colored metal inlay restoration, GCMC: gold-colored complete metal crown, GIn: gold-colored metal inlay restoration, RFMCBr: fixed partial dentures of RFMCs, RFMC: resin facing metal crown, PFMC: porcelain fused to metal crown, Br: fixed partial denture of CMCs, PFMCBr: fixed partial denture of PFMCs, CR: composite resin filling, and CC: ceramic crown.

At least 104 instances of each type of dental prosthesis and restoration were detected in the oral photographic images; the most common type was CMC (2147 instances) and the least common was RFMCBr (104 instances; Fig. 1). The number of prostheses classified as TP and FP are shown Fig. 2. The ratio of TP ranged from 0.67 in CR to 0.96 in CMC (Fig. 3). The APs of each dental prosthesis and restoration are as follows; CMC: 0.93, MIn: 0.92, GCMC: 0.90, GIn: 0.90, RFMCBr: 0.81, RFMC: 0.78, PFMC: 0.78, Br: 0.77, PFMCBr: 0.77, CR: 0.61, and CC: 0.59 (Fig. 4). The mAP and mIoU of this detection system are 0.80 and 0.76, respectively.

## Discussion

The proposed system will be developed by the computer itself, and basic information about the intraoral condition, like the prosthetic situation of the residual teeth, could be detected by the computer. In this study, the automated system which is identified prosthetic situation of residual teeth and distinguish between sound and restorative teeth from oral images were developed. This system will be a useful tool to make the treatment plans of each patient. On the other hand, further studies focused on caries risk and periodontal and endodontic situation using dental X-ray images will be necessary to grasp all the intraoral information.

When evaluating the performance of deep learning-based object detection, two indices, intersection over union (IoU) and mean average precision (mAP) were usually used whereas confusion matrix were generally used. An IoU of more than 0.7 is regarded as good^8,9^, and the IoU in this study is 0.76. Therefore, the performance of this learning system is high. A mAP is used to measure the accuracy of object detection model and A mAP of more than 0.7 seemed to be regarded as a good value in other studies^10^, but there is no clear criterion. The mAP of this study was reached at 0.80 and the performance of this learning system is also regarded as good from the point of view of mAP. The values of the hyperparameters and the ratio between train and test data were determined from the results of preliminary experiments with various combinations of their values. In the preliminary experiments, the IoU and mAP were almost unchanged among the situations, but learning using this combination yielded superior IoU and mAP values than other combinations.

YOLO was first reported in 2016^11^ as real-time object detection algorithm, modified version (YOLO 9000) was proposed in 2017^12^ and version 3 (YOLO v3) was proposed in 2018^13^. YOLO v3 was shown to have a detection performance that was higher than other detection algorithm such as SSD^14^ and RetinaNet^15^.

When selecting the dental prostheses and restorations to be detected in this study, the frequency of their occurrence in the images was considered. And sound teeth were not object of detection in this study, that is teeth which was not detected anything are sound teeth. Because, it is suitable for the object detection algorithm to detect something in particular, like metallic-colored restorations or prostheses in this study, but it is difficult to detect nothing in particular, like sound teeth. Moreover, those needed for selecting abutment teeth were also included. When selecting the abutment teeth, the shape and material of the prostheses and the filling area of the restorations should be considered. Therefore, eight prostheses and three restorations that frequently appear in the training images and are frequently used in daily clinical procedures were selected. Another limitation that determined the types of prostheses that were studied was the number of images. In this study, about 1900 images were used, but this number was not sufficient to recognize all kinds of prostheses used in dental clinical procedures. Therefore, if more kinds of prostheses are to be identified, more images of those types will be necessary.

The results of this study demonstrated that the AP of each dental prosthesis and restoration varied from 0.59 (for CCs) to 0.93 (for CMCs) with mAP of 0.80. Moreover, the ratios of correct detections also varied from 0.67 (for CRs) to 0.96 (for CMCs). That is, more than 90% of metallic-colored prostheses such as CMCs, GCMCs, MIns, and GIns, more than 80% of mixed-colored prostheses such as RFMC and PFMC or splinted prostheses like Brs, and about 70% of tooth-colored prostheses such as CCs and CRs could be detected when using this system. Regardless of the total number of objects in all images, the APs tended to be higher for metallic-colored prostheses and tended to be lower for tooth-colored prostheses. These results indicate that the detections were made according to the color differences between the teeth and prostheses.

In this study, two types of mistake occurred: one is misrecognition, in which a prosthesis was recognized as another type of prosthesis, and the other is misdetection, in which the prosthesis itself could not be detected. Misrecognition between similarly colored different prosthesis, like RFMC and PFMC, also occurred in some cases. This mistake occurred because the recognition is mainly based on the color difference. In fact, discrimination between similar sizes and different color prostheses, such as CMC and GCMC or MIn and GIn, was correct in most cases. Discrimination between similarly colored but different sized objects, such as MIn and CMC or GIn and GCMC, was also correct in most cases. These prostheses differ in size, are partially filled or fully covered, and have a combination metallic color and tooth color, which indicates the proportion of prosthesis or restoration in teeth. It is thought that discrimination among similarly colored prostheses used this combination of differences.

The recognition of splinted and unsplinted prostheses, for instance, unsplinted adjacent RFMCs and RFMCBr, was also correct about 80% of the time. Therefore, this system could recognize the difference in the shape around the splinting section of splinted and unsplinted prostheses. The results of the recognition using this system indicate that it can correctly detect the difference in shape at a high rate even if the difference is small. However, in the case of color differences, big differences such as those between metal and teeth could be recognized correctly even in small areas, but small differences such as those among ceramic, composite resin, and teeth could not be recognized with high accuracy. As a result, tooth-colored dental prostheses and restorations were misdetected. The misdetection of CCs and CRs in this system is frequent. In these cases, the CCs and teeth esthetically restored by CR were misdetected and recognized as sound teeth. On the other hand, MIns, GIns, and discolored CRs were detected with high accuracy even if they were very small. This result suggests that misdetection is caused not by small size but by similar color. This misdetection likely to happen and they sometimes cannot be told the difference even if seen by the human eyes. How to prevent this misdetection seems to be a big future issue of this system.

The result of this study indicates that the recognition of dental prostheses and restorations from the occlusal view of oral photographic images is highly accurate for those that are metallic in color. However, recognition is difficult for tooth-colored prostheses and restorations if only oral photographic images are used. Therefore, a combination other view of oral photographic images and other images, such as X-ray and digital scanning images, may be necessary for decreasing misrecognitions and misdetections. To improve the learning performance, more images of dental prostheses and restorations, especially of CCs and CRs, and image screening, which is the selection of images suitable for object detection, will be necessary.

Within the limitations of this study, the following conclusions were drawn.

1. Dental prostheses and restorations can be recognized from oral photographic images with mAP of 0.80 and mIoU of 0.76.
2. Dental prostheses and restorations with metallic color can be recognized with an AP of more than 0.80, but those with tooth color are recognized with an AP of about 0.60.
3. About 80% of dental prostheses and restorations with metallic color can be recognized correctly. This value is about 60% for those with tooth color.

## Material and methods

### Data collection

A total of 1904 oral photographic images (1084 maxillary and 820 mandibular images) were obtained from patients who attended Department of Prosthodontics, Gerodontorogy and Oral Rehabilitation of Osaka University Dental Hospital. They were occlusal view of whole arch and taken by dentists in charge using their own digital single-lens reflex cameras. All images were JPEG files that were resized to 416 × 416 pixels. This study protocol was approved by the Ethical Review Board at Osaka University Dental Hospital (H30-E26).

### Dental prosthesis and restoration annotation

Eight types of dental prostheses were annotated manually in all oral images using a graphical image annotation tool (labelImg^16^). They were silver-colored complete metal crowns (CMCs), gold-colored complete metal crowns (GCMCs), resin facing metal crowns (RFMCs), porcelain fused to metal crowns (PFMCs), ceramic crowns (CCs), and three kinds of fixed partial denture, CMCs (Br), RFMCs (RFMCBr), and PFMCs (PFMCBr). Three types of dental restoration were also annotated: composite resin fillings (CR), silver-colored metal inlay restorations (MIns), and gold-colored metal inlay restoration (GIns).

**Figure 5:**
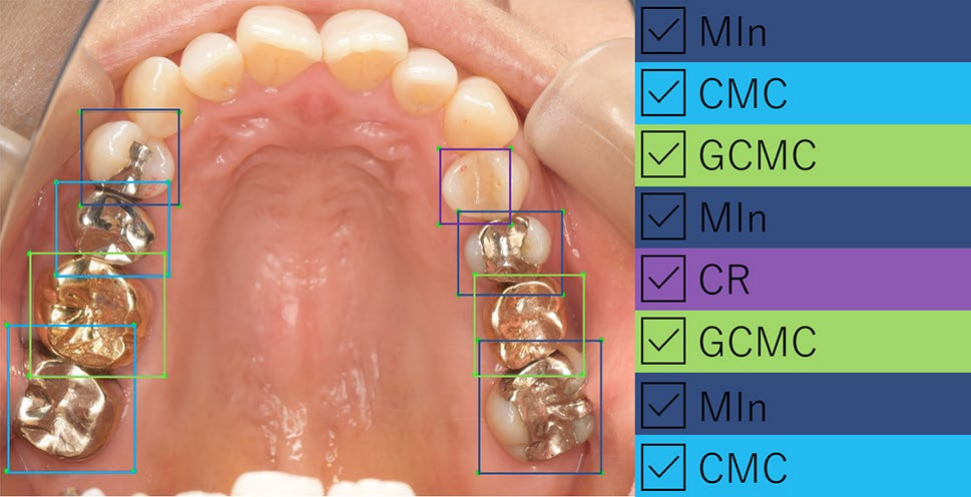
Sample image of annotation. Annotation was conducted using occlusal view of whole dental arch in each photograph image. MIn: silver-colored metal inlay restoration, CMC: silver-colored complete metal crown, GCMC: gold-colored complete metal crown and CR: composite resin filling.

The annotation was conducted using the image of whole dental arch. A sample image of annotation result was shown in Fig. 5. When annotating the prostheses or restorations, their types were decided based on the clinical record or the dental lab order form of each patient.

### Deep-learning algorithm

The images were randomly divided into two datasets: one for training (1524 images: 867 maxillary and 657 mandibular) and one for testing (380 images: 217 maxillary and 163 mandibular). To implement the object detection algorithm, Python 3.5.2 and the Keras library 2.2.4 was used with TensorFlow 1.12.0 as the backend. The object detection application, You Only Look Once version 3 (YOLOv3)^13^, with fine tuning was used and the dataset was trained to detect dental prostheses and restorations. The training dataset was separated into eight batches for every epoch and 5,000 epochs were run with a learning rate of 0.01.

### Assessment of the learning result

**Figure 6:**
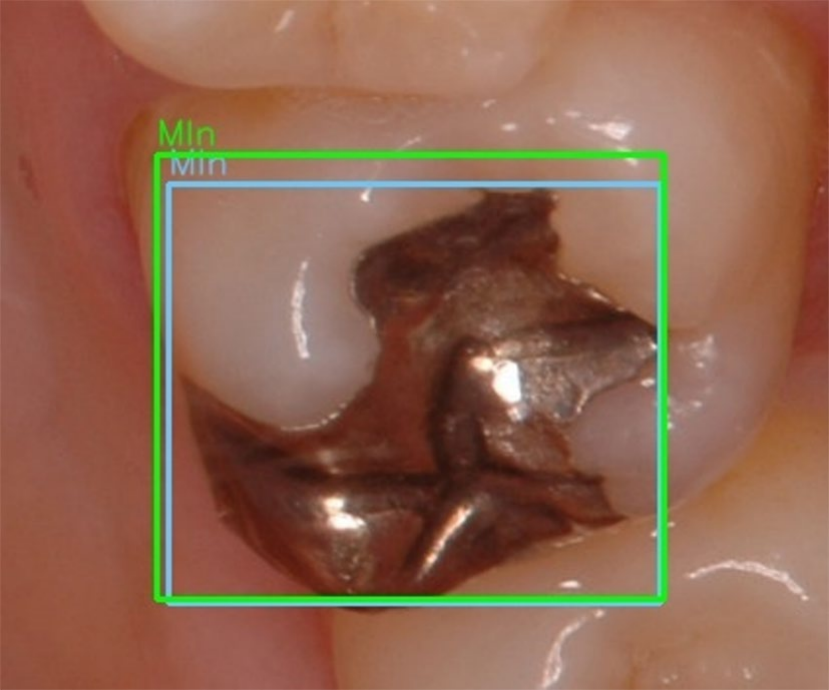
Sample image from the image recognition dataset containing a silver-colored inlay restoration (MIn). The green square indicates the ground-truth bounding box and the blue square indicates the predicted bounding box.

For all objects in all dataset images, the number of objects recognized correctly (true Positives; TP) and those recognized as other types of objects (false Positives; FP) were identified. The average precision (AP) of each type of dental prosthesis and restoration, the mean average precision (mAP) under an intersection-over-unit (IoU) of more than 0.5, and mean IoU (mIoU) were calculated. IoU is the ratio, ranging from 0 to 1, of the overlapping area of the ground-truth and predicted areas to the total area. It measures how closely the predicted area fits the ground-truth area. IoU was calculated according to the following formula. A sample image of calculating IoU was shown in Fig. 6.

$${\text{IoU}} = {\text{area of overlap }}\left( {{\text{both ground}} - {\text{truth bounding box and predicted bounding box}}} \right)/{\text{area of union }}\left( {{\text{either ground}} - {\text{truth bounding box or predicted bounding box}}} \right)$$

AP is the value, from 0 to 1, of the area under the Precision-Recall curve. The value of AP varies depending on the IoU threshold. In this study, the IoU threshold was set to 0.5, which is commonly used in other object detection studies^10^. The mAP is calculated by taking the average of AP over all classes. Higher values indicate more accurate learning.

### Ethical declarations

The study protocol was approved by the Ethical Review Board at Osaka University Dental Hospital (H30-E26). Informed consent was obtained from all participants.

### Ethical approval

All clinical procedures were performed in accordance with the ethical standards of the 1964 Helsinki declaration and its later amendments or comparable ethical standards.

### Informed consent

Informed consent was obtained from all individual participants included in the study.

## Data Availability

The datasets generated during and/or analysed during the current study are not publicly available due to protection of personal information and limitation of ethical approvement, but are available from the corresponding author on reasonable request.
